# Hereditary Spherocytosis: Can Next-Generation Sequencing of the Five Most Frequently Affected Genes Replace Time-Consuming Functional Investigations?

**DOI:** 10.3390/ijms242317021

**Published:** 2023-11-30

**Authors:** Friederike Häuser, Heidi Rossmann, Anke Adenaeuer, Annette Shrestha, Dana Marandiuc, Claudia Paret, Jörg Faber, Karl J. Lackner, Bernhard Lämmle, Olaf Beck

**Affiliations:** 1 Institute of Clinical Chemistry and Laboratory Medicine, University Medical Center of the Johannes Gutenberg University, 55131 Mainz, Germany; 2 Transfusion Center, University Medical Center of the Johannes Gutenberg University, 55131 Mainz, Germany; 3 Department of Pediatric Hematology, Oncology & Hemostaseology, Center for Pediatric and Adolescent Medicine, University Medical Center of the Johannes Gutenberg University, 55131 Mainz, Germany; 4 Center for Thrombosis and Hemostasis (CTH), University Medical Center of the Johannes Gutenberg University, 55131 Mainz, Germany; 5 Department of Hematology and Central Hematology Laboratory, Inselspital, Bern University Hospital, University of Bern, 3010 Bern, Switzerland; 6 Haemostasis Research Unit, University College London, London WC1E6BT, UK

**Keywords:** hereditary spherocytosis, NGS, RBC membrane disorder, *ANK1*, *EPB42*, *SLC4A1*, *SPTA1*, *SPTB*

## Abstract

Congenital defects of the erythrocyte membrane are common in northern Europe and all over the world. The resulting diseases, for example, hereditary spherocytosis (HS), are often underdiagnosed, partly due to their sometimes mild and asymptomatic courses. In addition to a broad clinical spectrum, this is also due to the occasionally complex diagnostics that are not available to every patient. To test whether next-generation sequencing (NGS) could replace time-consuming spherocytosis-specific functional tests, 22 consecutive patients with suspected red cell membranopathy underwent functional blood tests. We were able to identify the causative genetic defect in all patients with suspected HS who underwent genetic testing (*n* = 17). The sensitivity of the NGS approach, which tests five genes (*ANK1* (gene product: ankyrin1), *EPB42* (erythrocyte membrane protein band4.2), *SLC4A1* (band3), *SPTA1* (α-spectrin), and *SPTB* (β-spectrin)), was 100% (95% confidence interval: 81.5–100.0%). The major advantage of genetic testing in the paediatric setting is the small amount of blood required (<200 µL), and compared to functional assays, sample stability is not an issue. The combination of medical history, basic laboratory parameters, and an NGS panel with five genes is sufficient for diagnosis in most cases. Only in rare cases, a more comprehensive functional screening is required.

## 1. Introduction

Membranopathies affect the vertical or horizontal skeleton of the erythrocyte membrane. Because of congenital defects of the membrane proteins, red blood cells (RBC) show distinct morphological features, such as a spherical or elliptic shape. The destruction of the abnormal erythrocytes in the spleen is the main cause of the haemolytic anaemia seen in the majority of the patients affected [1]. Membranopathies are clinically, biochemically, and genetically heterogeneous [2], the severity of the haemolysis ranges from clinically insignificant to severe, life-threatening haemolytic anaemia [3]. They are typically caused by a quantitative or qualitative defect of an RBC membrane protein. Both vertical and horizontal membrane skeleton components may be involved.

The RBC membrane is highly elastic and resistible to fluid shear stress. It consists of a phospholipid bilayer and a two-dimensional network of cytoskeletal proteins [4,5]. There are different transmembrane complexes in the bilayer: The ankyrin complex, which is composed inter alia of the proteins ankyrin (gene product of *Ank1*), band3 (*SLC4A1*) and protein band4.2 (*EPB42*), and the junctional complex, which contains the protein 4.1 (*EPB41*). The components of the complexes fulfil a wide variety of functions, such as anion exchange (band3) [6,7], and are an integral part of the cytoskeleton. Further components of the structural network are both kinds of spectrin, α- and β-spectrin (*SPTA1*, *SPTB*). They form hetero-dimers, self-associate to tetramers and are connected horizontally by actin and the 4.1 junctional complex. The transmembrane ankyrin complex also binds to spectrin dimers and enables the vertical membrane anchoring of the cytoskeleton [1,5,8]. If binding within the ankyrin- or junctional complex, between the spectrin multimers themselves, or between the spectrins and the proteins of the complexes are weakened, or one of the involved proteins is not expressed sufficiently, the vertical or horizontal skeleton of the membrane is unstable or disrupted [9,10,11]. This results either in decreased mechanical integrity or in the loss of membrane and membrane cohesion [10,12]. Loss of surface area results in small, spherical erythrocytes packed with haemoglobin. Alteration in mechanical integrity is accompanied by permanent deformation under shear stress, membrane fragmentation, and the formation of elliptocytes [13]. Abnormally shaped RBCs are trapped in the splenic Billroth canals and phagocytosed by the reticuloendothelial system [14]. As a result, the severity of the disease depends on the extent of surface loss and ranges from asymptomatic to severe neonatal or even prenatal haemolytic disease [15].

The variants in the genes that are mainly responsible for the vertical membrane anchoring of the cytoskeleton (*ANK1*, *EPB42*, *SLC4A1*, *SPTA1*, and *SPTB*) are the cause of hereditary spherocytosis (HS). The variants in the gene encoding protein 4.1 (*EPB41*) and in the specific regions of the spectrin genes cause hereditary elliptocytosis (HE). HE and its aggravated form, the hereditary pyropoikilocytosis (HPP), are both characterized by a decreased horizontal stability of the cytoskeleton [13].

HS has the highest prevalence among Caucasians (1:2000), especially in northern Europe [1]. However, the frequency of the disease is probably underestimated because it is frequently asymptomatic or only very mild. More severely affected patients are characterised by neonatal jaundice, transfusion-dependent haemolytic anaemia in childhood, or gallstones. Moderately affected patients with compensated haemolysis are often diagnosed during an aplastic crisis, for example, during a parvovirus B19 infection [1].

HE has a worldwide prevalence of 1:2000–4000, but it is more common in malaria-endemic regions [13]. Similar to HS, the prevalence might be underestimated as HE is frequently asymptomatic or causes only mild haemolytic anaemia [11,13,16]. The consequence of homozygous or compound heterozygous HE variants is the more severe HPP [16,17].

Haemolytic anaemia is observed in most patients with RBC membrane disorder. However, due to the different molecular defects and affected genes, there is a wide range of disease severity, which modifies therapeutic management [11,18]. In severe forms of HS, splenectomy should be considered [19]; however, it is contraindicated in stomatocytosis due to possible thromboembolic complications [20]. Stomatocytosis is a defect of the membrane channel proteins Piezo1 or Gardos with similar symptoms as HS [9]; therefore, an accurate diagnosis is necessary in these patients to prevent false treatments. In addition, a reliable diagnosis is also relevant for the prediction of disease severity and family counselling, especially due to the variety of inheritance patterns.

**Figure 1:**
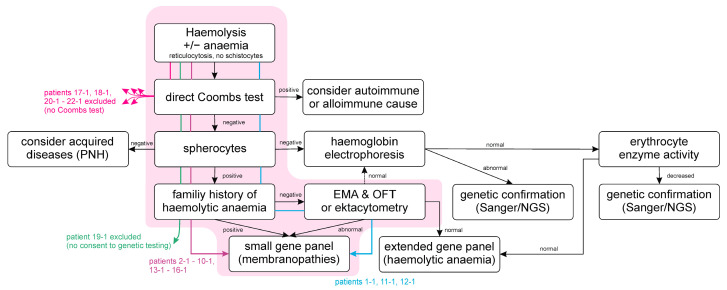
Diagnostic workup of patients with Coombs-negative haemolysis at the University Medical Center Mainz. Haemolysis was defined based on the laboratory findings of reticulocytosis, elevated LDH, elevated (unconjugated) bilirubin, and lowered haptoglobin. All index patients with a suspicion of spherocytosis in our centre between June 2019 and June 2023 are assigned by his/her patient number (for patient details see Table 1 and Table S3) in the flow chart. The algorithm for the inclusion of patients in the systematic evaluation of the diagnostic procedure is highlighted in red (see also the figure of Section 2.2.1). Patients who did not meet these criteria were excluded from these evaluations (patients 17-1–22-1). EMA = eosin-5-maleimide binding test, OFT = osmotic fragility test, PNH = Paroxysmal nocturnal haemoglobinuria.

For the diagnosis of RBC membrane disorders, the guidelines recommend a step-by-step approach [21,22], as shown in Figure 1. In addition to clinical symptoms and family history, basic laboratory tests (haemolysis parameters, complete blood count (CBC) and detection of spherocytes in a blood smear) are the first diagnostic steps. Functional tests (eosin-5-maleimide test (EMA), fragility tests of the RBC or ektacytometry) are used to confirm the diagnosis. Functional tests require fresh sample material and must be performed on the same day or, in the case of ektacytometry, within 72 h. The shipment of samples for this type of analysis to a specialised laboratory often results in compromised quality of the sample. Genetic confirmation of the findings is not recommended as standard procedure in the guidelines. In the past, the number of genes potentially involved and their size have made molecular diagnosis difficult. Sanger sequencing is of limited value in diagnosing complex disorders with locus heterogeneity. In these cases, Sanger sequencing is costly and time-consuming. On the other hand, next-generation sequencing (NGS) offers a time- and cost-effective approach to the molecular diagnosis of hereditary haemolytic anaemia. Especially in large diagnostic laboratories with many samples, NGS is easier to perform than functional tests. Thus, the diagnostic paradigm of congenital haemolytic anaemias is changing nowadays.

In a retrospective study, we included all patients (*n* = 16) of the Department of Pediatric Hematology and Oncology of the University Medical Center Mainz presenting between June 2019 and June 2023 with Coombs-negative haemolysis, spherocytes (blood smear), and a positive family history for haemolytic anaemia or at least positive EMA and/or OFT (osmotic fragility test). We analysed the diagnostic significance of a typical RBC morphology (detected by spherocytes, CBC, EMA, OFT) and performed NGS on the five most frequently affected genes for the final diagnosis. We focused on the question whether NGS can replace time-consuming spherocytosis-specific functional testing requiring fresh material (EMA, OFT) without reducing sensitivity or specificity.

In a second step, we retrospectively analysed the NGS results of all patients with a request for molecular genetic analysis for HS in the Institute of Clinical Chemistry and Laboratory Medicine of the University Medical Center Mainz. The collective consisted of the 16 aforementioned patients and 2 who did not meet the inclusion criteria for the first approach (total: 18). In a first step, only the five most frequently affected genes *ANK1*, *EPB42*, *SPTA1*, *SPTB,* and *SLC4A1* were considered. To ensure that no causative variants were missed, we subjected all samples to a further evaluation taking into account 117 anaemia-relevant genes. The aim was to characterize the variant/gene spectrum in a local collective, elucidate the mode of inheritance in the families (autosomal dominant, recessive, de novo) and address the question whether causative variants may be missed by the five-gene approach.

## 2. Results

### 2.1. Exemplary Case Report

**Figure 2:**
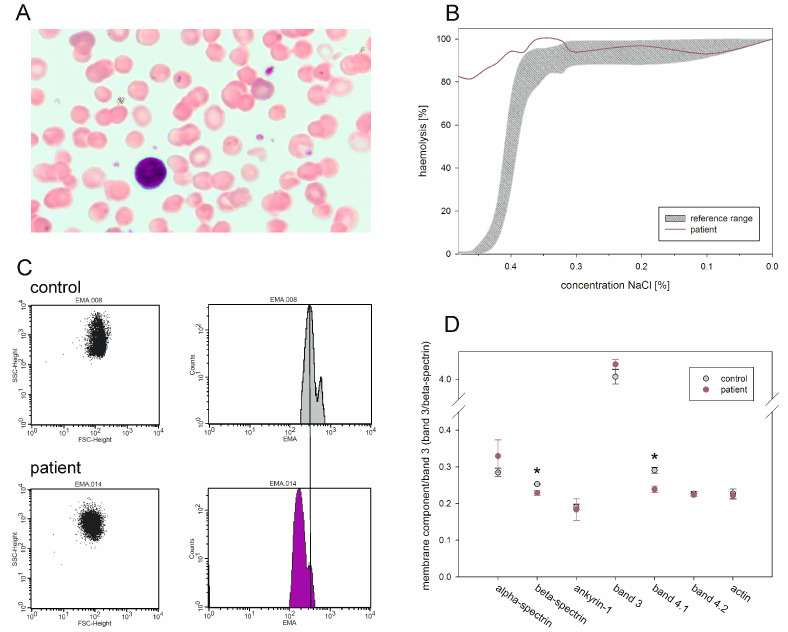
Laboratory results of patient 12-1, harbouring the nonsense variant p.(Gln1587Ter) in *SPTB*. (**A**) Exemplary peripheral blood smear with one leukocyte and numerous small, hyperchromic erythrocytes. (**B**) Osmotic fragility of the patient´s red blood cells compared to a laboratory reference range (grey). Over 80% of the patient’s erythrocytes already show haemolysis at a NaCl concentration of 0.48%. (**C**) Patient´s erythrocytes show reduced staining with EMA (left shift of the relative fluorescence, lower right panel) compared to the erythrocytes of a healthy donor (upper right panel). EMA staining of erythrocytes was quantified on a FACSCanto in 10,000 cells measured in triplicate. The representative result of one measurement is shown. (**D**) Densitometry results of the SDS-PAGE of erythrocyte membrane proteins from the patient and three healthy donors, measured in triplicates. The density of the membrane protein components was compared to the density of band3 (band3 was compared to the density of β-spectrin). β-spectrin and the protein band4.1 (which binds to the spectrins in vivo) are lower than the controls (* *p* < 0.001, Welch´s *t*-test). For the remaining proteins, no significant difference was observed, as determined by Welch´s *t*-test.

The exemplary case report illustrates our algorithm for the diagnosis of an RBC membrane disorder, as shown in Figure 1, which is in agreement with current diagnostic guidelines. At the age of nine, patient 12-1 presented at the Center for Pediatric and Adolescent Medicine of the University Medical Center Mainz. She had suffered from recurrent anaemia. In the past, she had had several aplastic crises in addition to the first one, observed at the age of four years, associated with parvovirus B19 infection. She received multiple blood transfusions at the age of three and five years. At the time of presentation in our centre, CBC revealed no anaemia (haemoglobin 12.8 g/dL) but reticulocytosis (8%, 332 reticulocytes/nL). Red blood cells were microcytic, which was indicated by MCV (79 fL) and the percentage of microcytes (11%). In total, 42% of hyperchromic erythrocytes were detected. MCHC was elevated (37.9 g/dL; see also Table S1). Spherocytes were observed in blood smear (Figure 2A). Besides the reticulocytosis, the results of clinical chemistry suggested haemolysis. LDH and (unconjugated) bilirubin were elevated, and haptoglobin was below the limit of detection. An alloimmune or autoimmune cause of haemolysis was ruled out by a negative direct Coombs test (DCT). Therefore, an RBC membrane disorder was suspected. As neither the father nor the mother of the patient had a history of haemolysis, OFT and EMA were carried out. The osmotic fragility of the RBC was increased (Figure 2B), and a reduced binding of EMA in the fluorescence-based flow cytometric test compared to healthy controls could be observed (Figure 2C). The diagnosis of spherocytosis was made. Due to the anamnestic aplastic crises that required blood transfusions and the clinical sequelae of haemolysis (hepatomegaly/cholelithiasis), which had aggravated over time, the disease severity was assessed as severe.

To confirm the diagnosis, the patient and her parents underwent genetic testing. In addition, the SDS-PAGE of RBC membrane proteins was performed. This showed a reduced proportion of the membrane components β-spectrin and protein 4.1 (Figure 2D). The result of the SDS-PAGE corresponds well to the genetic result: a heterozygous nonsense variant in *SPTB* coding for β-spectrin (c.[4759C>T];[=], p.[(Gln1587Ter)];[=]). The CBC of the patient´s parents were inconspicuous, and no spherocytes were detected in their blood smears. The truncating *SPTB* variant detected in the proposita was absent in her parents (c.[4759C=];[4759C=], p.[(Gln1587=)];[(Gln1587=)]), suggesting a de novo occurrence. According to the ACMG/AMP (American College of Medical Genetics and Genomics and the Association for Molecular Pathology) guidelines [23], we classified the variant, which was assessed as “deleterious” by the in silico prediction tools, and which was detected in *SPTB*, a gene known for its association to spherocytosis, as “pathogenic”. Thus, spherocytosis type 2 (OMIM # 616649) was diagnosed.

At the age of seven and nine years, the patient was treated in our centre due to a developing inflammatory bowel disease (IBD). While therapy for IBD has focus, the burden of spherocytosis, such as choledocholithiasis and anaemia, is less severe.

### 2.2. Clinical Characteristics of the Patients Included in the Retrospective Evaluation of Laboratory Tests for HS

All included patients (1-1–16-1, criteria: see Methods section) had a strong clinical suspicion of erythrocyte membrane defect and showed at least one typical symptom (aplastic crisis, hepato-splenomegaly, gallstones, prolonged jaundice at birth, or one or more blood transfusions in the past [11,24,25]. The clinical severity of the disease was determined according to the German spherocytosis guidelines [24,25]. It ranged from light (*n* = 4) to severe (*n* = 5), most of the patients (*n* = 6) were moderately affected. The severity could not yet be assessed in the 2-month-old patient 6-1. Ten patients had already been affected by an aplastic crisis, while five showed only chronic haemolysis with transient anaemia. The detailed characteristics of the patients’ medical history can be found in Table S2. In 12 cases, we were also able to examine family members of the index patient. These results are also listed in Table S2.

#### 2.2.1. Haematological Characteristics of the Included Patients (1-1–16-1)

**Figure 3:**
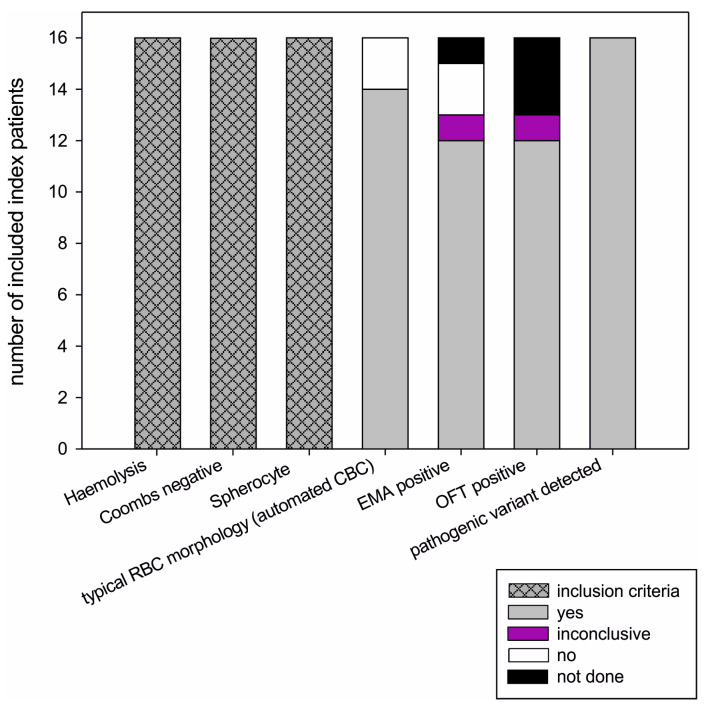
Laboratory-diagnostic results obtained from the 16 included patients (inclusion criteria: see Figure 1). OFT and EMA, as well as typical red blood cell morphology in automated blood cell count (CBC: MCV↓, MCHC↑, or hyperchromatic microcytes↑) were not positive/conclusive in all patients. However, a genetic cause of membranopathy was detected in all patients with Coombs negative haemolysis and spherocytes.

All 16 patients suffered from haemolysis (defined by at least two of the following criteria: LDH↑, haptoglobin↓, bilirubin↑, reticulocytosis) in nine patients accompanied by anaemia. Spherocytes and a negative DCT were part of the inclusion criteria and were present in all patients. OFT, EMA and SDS-PAGE were not available for all patients (Figure 3, Table S3). OFT was performed in 13 index patients, and fragility was increased in twelve. The result in patient 14-1 was inconclusive, but the osmotic fragility of his sister’s RBCs (family member 14-2) was abnormal. In 15 patients, the EMA binding test was performed. EMA binding was significantly reduced in 12 patients. The result of patient 14-1 was again inconclusive (with a pathologic result in his sister). Only patients 3-1 and 16-1 showed normal binding of EMA to band3, whereas osmotic fragility was increased in patient 3-1. CBC was performed in 21 relatives, and haemolysis was determined in 8 relatives. In all of the eight tested relatives, the result of the DCT was negative. In seven relatives, an osmotic fragility test was performed (pathological results in four individuals). In addition, EMA binding was tested in four relatives (EMA was pathologic in one individual). RBC membrane proteins were separated by SDS-PAGE in one relative and showed decreased spectrin fractions. An overview of the results can be found in Table 1. The detailed laboratory results are listed in supplemental Table S3. Data shown were obtained a maximum of 2 weeks before or after measurement of osmotic fragility and EMA test. This corresponds to the time of laboratory diagnosis.

#### 2.2.2. Haematological Characteristics of the Excluded Patients (17-1–22-1)

Initially, we included all patients with the suspicion of a RBC membrane disorder, who presented to the Center for Pediatric and Adolescent Medicine of the University Medical Center between June 2019 and June 2023 (*n* = 22, 1-1–22-1, Table 1, Tables S2 and S3). According to the diagnostic workup illustrated in Figure 1, the following six individuals with a diagnosis of haemolysis were excluded from systematic evaluation of the laboratory tests: Patients 17-1 and 18-1 due to the lack of a Coombs test. Nevertheless, sequencing had been performed in these two cases in the context of routine diagnostics. From patients 19-1 to 22-1, all who showed signs of an erythrocyte membrane disorder, consent to genetic testing had not been obtained at their first visit. They were then lost to follow-up. Therefore, family history and laboratory results were obtained incompletely, and no genetic testing was performed. Their data are presented in Table S3.

#### 2.2.3. Evaluation of NGS Results (1-1–18-1)

In addition to the 16 included patients, 2 further patients (17-1 and 18-1) were genetically tested. The resulting collective represents all index patients with the request for molecular genetic testing for an RBC membrane disorder from the Center for Pediatric and Adolescent Medicine of the University Medical Center Mainz within the study period. Except for patient 18-1, a disease-causing genotype was found in all index patients (Table 1). At her first visit, she had presented with haemolysis, borderline elevated MCHC, slightly increased hyperchromic RBCs, and an inconclusive blood smear. The EMA test was negative, a DCT was not performed, and molecular genetic testing was initiated. At the second visit, however, her haemolysis-related laboratory tests were in the reference range making a genetic cause very unlikely.

**Figure 4:**
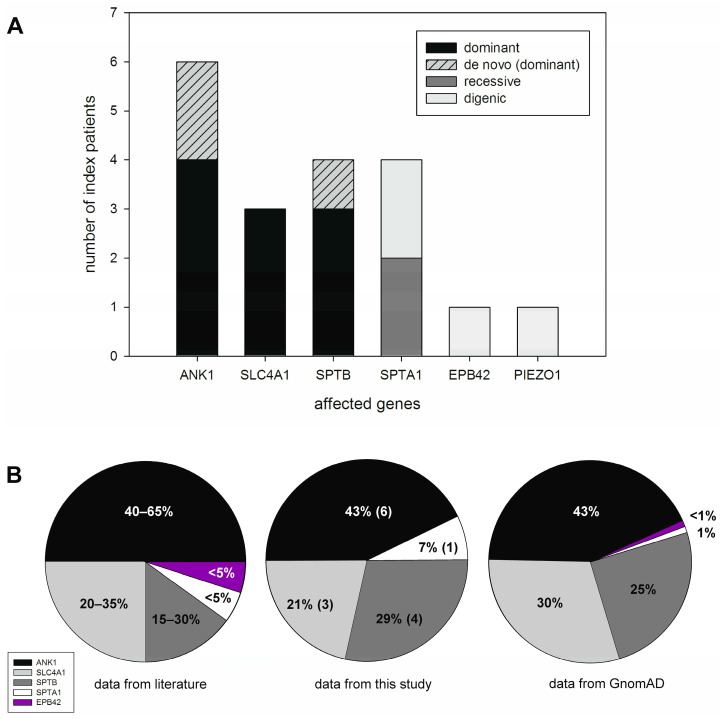
Inheritance pattern and frequency of the affected genes detected in the 17 patients analysed for the genetic cause of a membranopathy in our laboratory between June 2019 and June 2023 as well as data from the literature and database. (**A**) In 13 patients, the inheritance pattern was dominant, with 3 variants emerging de novo. Paternity has not been verified. However, the genes and variant types are consistent with those described in the literature as typical for de novo variants. The variant segregated with the phenotype or family history suggested a dominant inheritance in the remaining 10 patients. The inheritance pattern was recessive in two patients, with all variants occurring in *SPTA1*. In two patients, we detected a complex allele in *SPTA1* and a further missense variant in *EPB42* or *PIEZO1*, respectively. In these cases, we assume a digenic cause. (**B**) Similar frequencies of HS variants in the affected genes can be observed in data from the literature [9,26], in our collective, and in data calculated from GnomAD. Only HS patients with monogenic inheritance were considered (*n* = 14), the hereditary pyropoikilocytosis patient (Index-patient 3-1) and the patients with a digenic inheritance pattern (patient 6-1 and 16-1) were not included in the chart.

Unequivocally pathogenic variants were found in just the following four genes: *ANK1*, *SLC4A1*, *SPTA1*, and *SPTB*. In two patients with potentially digenic transmission, two likely pathogenic variants were detected in *EPB42* and *PIEZO1*. A total of 20 variants (including the two low-expression alleles) were found (Table 2, Figure 4A). Twelve of these variants are novel. In 13 patients, autosomal dominant pathogenic variants causing HS (OMIM # 182900, # 616649, # 612653) were detected. Patient 3-1 is compound heterozygous for two variants in *SPTA1*, causative for HPP (OMIM # 266140). A further patient is also compound heterozygous for a low expression variant (patient 15-1: αLEPRA (and αLELY)) and a null variant in *SPTA1*, as it is described for HS (OMIM # 270970). In patients 6-1 and 16-1, the transmission is potentially digenic because an additional variant was detected in *PIEZO1* and *EPB42*, respectively. The *PIEZO1* variant was the only one called in the second NGS analysis step, the remaining 19 variants were detected during the first filtering. Family analysis (genetic testing and family history) suggests de novo variants in three patients (1-1, 11-1, 12-1). In six patients, the incomplete availability of samples from family members hindered conclusive segregation analysis. Most variants were detected in *ANK1* (four new variants and two previously described ones), followed by the spectrin genes. Additionally, one new variant and the previously described variant Bicêtre I were found in *SLC4A1*.

All detected, presumably pathogenic variants are listed in Table 2, including the results of in silico analyses, the classification according to ACMG/AMP [23], and the references, if previously described. Further variants that probably do not contribute to the clinical symptoms (for example αLEPRA in a patient with a pathogenic *ANK1* variant) are listed in Table S4. The variant genes and the inheritance pattern are shown in Figure 4. The position of the variant in the respective gene is annotated in Figure S1.

In six patients (1-1, 4-1, 8-1, 11-1, 13-1, 16-1), previously described pathogenic variants were detected (Table 1 and Table 2). In 12 patients, the following new disease-causing/likely disease-causing variants were detected: three nonsense variants (in patients 9-1, 12-1, 14-1), two frame shift variants (5-1 and 17-1), two variants located in canonic splice sites (7-1 and 15-1), one variant predicted to cause a splice defect (10-1), and four missense variants (2-1, 3-1, 6-1, 16-1). The variants are all located in known spherocytosis/elliptocytosis-associated genes and are either absent from the international variant aggregating databases (dbSNP, ClinVar, GnomAD) or extremely rare (Table 2). The second variant calling step (117 anaemia-related genes) identified an additional likely pathogenic variant in *PIEZO1* in patient 6-1. This is the only variant that was not detected during the first variant calling step.

## 3. Discussion

The retrospective study of 16 patients with RBC membrane defects and their families over a period of 5 years was designed to answer the question of whether standard laboratory tests are sufficient for screening prior to genetic testing instead of time-consuming functional tests. It also aimed to determine the minimum number of genes that need to be investigated to make a genetic diagnosis in these patients. In addition, the spectrum of the variants and mode of inheritance of RBC membrane defects was investigated in a total of 18 patients.

### 3.1. Genetic Testing Characterised All Patients Correctly

In total, 18 index patients were genetically evaluated, who represent all genetically tested patients with suspicion of HS presenting at the Center for Pediatric and Adolescent Medicine of the University Medical Center within the study period. All 18 patients underwent a targeted exome sequencing; however, the initial variant calling was limited to five genes (*ANK1*, *EPB42*, *SLC4A1*, *SPTA1*, *SPTB*). By analysing the five spherocytosis-relevant genes, causative variants were detected in all patients with the diagnosis of an RBC membrane disorder. During the second variant calling step, a further variant in *PIEZO1* was detected in one patient (6-1). This fits well with his erythrocyte morphology (Figure S2). The variant was not detected in the five-gene approach. However, if, as in this case, the phenotype and genotype are not congruent (6-1 is a carrier of a complex allele in SPTA1 without any other variant in this gene), extended diagnostics should follow. Only in patient 18-1 no causative variant was found. In this case, however, HS was unlikely because the initially observed standard laboratory values had resolved at the second visit.

A total of 20 variants were detected in the tested index patients. Beside the well-described variant “band3 Bicêtre I” in *SLC4A1* [30], we observed seven further previously described or ClinVar (https://www.ncbi.nlm.nih.gov/clinvar/, accessed on 14 June 2023) listed variants, most of them in *ANK1*, followed by *SPTB*, *SLC4A1*, and, to a minor extent, in *SPTA1* and *EPB42* (Figure 4A). Despite the small number of individuals studied, the frequency of affected genes in HS corresponds to that described for the Caucasian collective in the literature [9,26] and that calculated from the genome aggregation database GnomAD (https://gnomad.broadinstitute.org, accessed on 19 October 2023, Figure 4B).

Numerous variants have been described in the past few years due to massive parallel sequencing possibilities [34,35,36]. The number of newly detected/private variants, also in this work, underlines the heterogeneity of the causative variants. Nevertheless, the number of causative genes remains limited: For HS, the variants are nearly exclusively found in the five genes *ANK1*, *EPB42*, *SLC4A1*, *SPTA1*, and *SPTB*. Hence, investigating a large number of genes does not improve test sensitivity in a collective with clinically relevant HS. In the literature, as well as in this study, causative variants were detected in one of the five genes in nearly all cases of HS (literature: 86–100%) [16,35,37,38,39,40]. As expected, sensitivity decreases if the diagnosis is more broadly defined, for example, as Coombs-negative chronic haemolytic anaemia in general [3,41,42].

We propose that the read-out of a small multigene panel containing only the established HS-causing genes is sufficient for the genetic characterisation of the majority of the patients. Regardless of whether a small panel of a few genes or a large panel or even an exome is sequenced, a stringent read-out avoids unnecessary data and facilitates straightforward interpretation. This approach results in a small number of variants of unknown significance (VUS), which would otherwise have to be additionally classified and reviewed. In addition, in some countries, such as Germany, only the read-out of clearly disease-associated genes is permitted if such can be defined. If another form of membrane defect is suspected from the blood smear or the co-inheritance of a membranopathy with a further congenital defect of the RBC (for example, sickle cell trait or glucose-6-phosphate dehydrogenase deficiency) is assumed, the read-out of the small spherocytosis panel can be extended. However, a small panel is only useful if there is a high pre-test probability for the diagnosis of HS.

### 3.2. Evaluation of Analytical Methods for HS That Provide a Reasonable Pre-Test Probability for Performing NGS

In order to investigate which laboratory tests and clinical findings are suitable to assure a sufficient pre-test probability, 16 index patients with defined inclusion criteria (Figure 1) suggesting an RBC membrane defect and their families (38 individuals) were tested functionally and genetically.

In addition to blood count, blood smear, haemolysis parameters, and DCT, guidelines recommend HS-specific testing to confirm the diagnosis, either the combination of a RBC fragility test (acidified glycerol lysis test (AGLT), OFT) with the EMA or, alternatively, ektacytometry [21,25,43]. In our diagnostic routine, OFT and EMA are established. In general, this combination shows good sensitivity and specificity (95%), although the sensitivity of AGLT and EMA was suggested to be slightly higher [2,44]; however, none of these tests can detect all HS cases [2]. A negative result in OFT does not rule out an erythrocyte membrane defect (sensitivity 81%) [44]. In our study collective of 16 patients (Figure 3), EMA and OFT were both inconclusive in 1 patient each, and EMA was negative in 2 patients. In contrast, in all 16 patients an RBC membrane disorder-causing genotype was found. SDS-PAGE was recommended to complement diagnostics [43], but the method is tedious and time-consuming. Furthermore, in the network of proteins maintaining RBC shape, a decrease of one protein impairs the concentration of its binding partners; therefore, the quantification of RBC membrane proteins by SDS-PAGE is often difficult to interpret [45]. Osmotic gradient ektacytometry is the reference test for cell membrane disorders [46], but the disadvantage is the need of specialised equipment and therefore the limited availability of this technique. Even EMA, OFT, AGLT, and SDS-PAGE are not offered by all laboratories because they require special equipment (for example, fluorescence flow cytometer), or, if instrumentally simple, elaborate preparations (RBC membrane ghosts for PAGE). In any case, fresh material is needed; therefore, experienced personal must be available to carry out the investigation on-site in a timely manner. A shipment of samples to a specialised laboratory is time and temperature critical and is a frequent cause of low-quality results.

Since NGS is part of the routine diagnostics in numerous laboratories and is pre-analytically uncritical, it can be an alternative to specialised testing. Only a small amount of material is needed (about 200 µL whole blood), and the material can be stored until or shipped for analysis without a loss of result quality. Thus, NGS is a time- and cost-effective alternative to conventional HS-specific tests. Recently, it has begun to replace standard methods for many diagnostic purposes, and diagnostics is moving towards personalised medicine through NGS [47].

A broad range from 11 to 70% sensitivity has been reported for different NGS-based approaches in patients with the diagnosis “haemolytic anaemia” [36,48]. Here, the sensitivity depends mostly on the number of genes analysed. In addition, a large number of variants of uncertain significance are detected in these patients, which cannot be classified correctly, inter alia, due to the lack of clinical data and routine laboratory analyses that provide clues for the cause of haemolysis. With appropriate phenotyping, the sensitivity of an NGS panel increases significantly (from 70 to 100%) [48]. The high sensitivity of NGS was confirmed in our HS collective: We were able to detect the genetic cause of the disease in all well-characterised, examined index patients (Figure 3) by primarily evaluating the five main HS-associated genes. Only in one patient, who had been excluded from the study according to the study criteria and later on turned out to be not affected by HS, molecular genetic testing was negative. Furthermore, our data suggest that appropriate phenotyping does not necessarily require complex laboratory analyses. Simple, but consequently and, if possible, at two different time points performed investigations are sufficient to provide a pre-test probability, justifying initiating a 5-gene NGS panel: (1) clinical presentation (phenotype and family history), (2) markers of haemolysis (e.g., haptoglobin, reticulocytosis), (3) DCT, (4) CBC, and (5) a blood smear to detect spherocytes. Our data suggest that a positive family history further increases pre-test probability by about 20–50% but reduces sensitivity significantly because patients with de novo mutations and recessive inheritance of the disease are excluded. Our concept is not to abolish EMA, OFT, AGLT and ektacytometry but to limit them to the probably rare, ambiguous cases. Prospective studies are needed to validate this new algorithm.

We only examined patients with a strong suspicion of HS according to the German S1 guidelines [21,25] and a workflow adapted from Risinger and Kalfa [11]. This also explains the high sensitivity in our collective. According to the guidelines, genetic testing is not necessary when the diagnosis can be made based on clinical history, family history and laboratory findings (haemolysis, elevated MCHC, detection of spherocytes, abnormal functional test (e.g., OFT, EMA, AGLT)) [21,25,43]. Genotyping of the patients is not specifically mentioned in these guidelines; however, it is becoming more and more a part of the recommendations [49]. In these days, neither the size and number of genes nor the price of the test significantly matter in NGS, especially compared to the technically complex functional testing; therefore, we recommend the genetic confirmation in any case. Even when examining patients with, for example, an empty family history but an otherwise clear diagnosis of membranopathy, where tests such as EMA or OFT are otherwise carried out, NGS can be performed instead.

Even though recommended by the guidelines only in the case of uncertainty, we believe that genetic characterization is important for all patients with clinically relevant HS in order to facilitate a rough estimate of the course of the disease, possible treatment options (splenectomy yes/no), and, above all, to enable genetic counselling for patients and their families. This is particularly interesting in the case of RBC membrane disorders as inheritance can follow both a dominant and recessive pattern, and numerous de novo variants have also been described. The *SPTA1* low expression variant αLELY, which occurs frequently in the European population, can also influence the phenotype (HE/HPP) [16] and should be considered in family counselling.

Of course, our screening strategy might miss mildly affected patients. Whether genetic testing of mildly affected individuals, who may be potential carriers of a severe recessive variant, and of patients with haemolysis of an unclear cause should be performed remains to be discussed. In these cases, unless truncating variants are detected, it may be difficult to define the causative variants among the candidates.

Depending on the equipment of the laboratory, we propose to adapt the diagnostic algorithm for HS to facilitate both, either to perform HS-specific functional tests (EMA, fragility tests, ektacytometry) first or, alternatively, NGS; thus, in patients with Coombs negative haemolysis and spherocytes, genetic testing can be the primary diagnostic option. The necessary standard tests (CBC (including MCHC, reticulocytes), blood smear, haemolysis parameters, DCT) can be carried out by almost any laboratory. Only in unclear cases (for example, genotype-phenotype inconsistency) further testing, such as EMA or ektacytometry, should be additionally performed. Nevertheless, it should be mentioned that in about 10% of patients the molecular cause remains unclear [10].

## 4. Materials and Methods

### 4.1. Patients

In total, 22 consecutive patients under the age of 18 years with the suspicion of a membranopathy (defined as follows: Coombs-negative haemolysis, spherocytes, and a positive family history for haemolytic anaemia or at least positive EMA and/or OFT) who presented to the Department of Pediatric Hematology, Oncology & Hemostaseology, Center for Pediatric and Adolescent Medicine of the University Medical Center Mainz between June 2019 and June 2023 were retrospectively included in this study (*n* = 22; 1-1–22-1). All patients were examined by the same paediatrician. The inclusion algorithm is shown in Figure 1.

Four (19-1–22-1) out of 22 patients were excluded because initially no consent to molecular diagnostics had been obtained and there was no further possibility to do so (loss to follow-up). Thus, 18 index patients and 22 of their relatives were genetically examined. Eleven out of 18 index patients were female, seven were male. The median age at clinical diagnosis was 3.5 years (one day–17 years); the median age at sample collection for molecular confirmation was six years (one day–18 years). For systematic evaluation of diagnostic tests, two further index patients (17-1, 18-1, both female) were excluded as no direct Coombs test was performed. Thus, 16 patients (1-1–16-1) were considered.

Besides laboratory analysis of the patients’ blood, all patients were physically examined and a personal and family history was obtained. In twelve cases, we were able to screen one to three relatives of the index patients. The age of the relatives given is that at the time of blood collection for segregation analysis, which is not identical to the time of diagnosis of the index patient.

All analyses were carried out for diagnostic purposes. All individuals consented to genetic testing in accordance with the German Genetic Diagnostics Act (GenDG) and explicitly agreed to pseudonymised analysis of their genetic results for scientific purposes.

### 4.2. Complete Blood Count and Clinical Chemistry Parameters

Routine laboratory parameters such as haptoglobin, lactate dehydrogenase (LDH), bilirubin (automated photometer/potentiometer Alinity c; Abbott Diagnostics, Wiesbaden, Germany), and complete blood count (CBC; automated Advia 2120i hematology system, Siemens Healthcare GmbH, Erlangen, Germany) were determined at every inpatient and outpatient clinic consultation. May-Grunwald-Giemsa-stained blood smears were also preparated by the autoslide component of the Advia 2120i.

### 4.3. Osmotic Fragility Test

The osmotic fragility of the erythrocytes was tested according to the protocol of Dacie and Lewis [50]. After 30 min incubation of anticoagulated blood with a series of progressively hypotonic saline solutions (0.9–0.0% NaCl in aqua dest.), haemolysis was determined in the supernatant at 546 nm by cyanmethemoglobin method (Drabkin’s reagent, 1:12.5; Sigma-Aldrich Chemie GmbH, Taufkirchen, Germany). The test was repeated after an 24 h incubation step at 37 °C.

### 4.4. Eosin-5-Maleimide Binding Test

EMA test was performed as described by King et al. [51]. The fluorescent dye eosin-5-maleimide binds to the intact membrane of RBC (predominantly to band3), and to a lesser extent to RBC with membrane defects. The discrepancy can be detected as lower fluorescence readings by flow cytometric analysis. In total, 5 µL of phosphate-buffered saline (PBS) washed RBC (1:50 (*v*/*v*); 1500 g, 5 min, RT) were stained with 25 µL EMA (0.5 mg/mL; Sigma-Aldrich) and incubated for 1 h at room temperature (RT) in the dark. In order to remove unbound dye, the labelled RBCs were washed three times with PBS/BSA (0.5% bovine serum albumin in PBS; 1:50 (*v*/*v*)). For flow cytometric analysis, the pellet was resuspended in PBS/BSA, the 1:7 diluted RBC were counted on a FACSCanto II (BD Biosciences, Heidelberg, Germany, 10,000 events/sample). EMA binding to patient erythrocyte membrane was compared to that of three controls.

### 4.5. Direct Coombs Test

The direct Coombs test (DCT) was performed at least once. The following three assays were used over the years: column agglutination on gel cards containing either polyspecific or monospecific anti-human globulin reagents (LISS/Coombs or DC-Screening I cards from Bio-Rad Laboratories GmbH, Feldkirchen, Germany), and the automated solid phase assay on Galileo Neo (Immucor Medizinische Diagnostik GmbH, Dreieich, Germany).

### 4.6. SDS-PAGE

EDTA blood was processed within two hours after collection (800 g, 10 min, RT). After the removal of plasma and leukocytes, PBS was used to wash erythrocytes twice (1:1 (*v*/*v*); 800 g, 10 min, RT). The RBCs were lysed on ice with 1:20 (*v*/*v*) modified hypotonic lysis buffer (1 mM EDTA, 5 mM Na_2_HPO_4_, pH 7.4) and washed four times with the lysis buffer (20,000 g, 40 min, 4 °C) [52]. The membranes were dissolved in reducing Laemmli sample buffer (62.5 mM Tris/Cl, pH 6.8, 2% SDS (sodium dodecyl sulfate, *w*/*v*), 10% glycerol (*w*/*v*), 5% 2-mercapto-ethanol (*v*/*v*), and 0.001% bromphenol blue (*w*/*v*)), aliquoted, and stored at −80 °C until electrophoretic separation on 10% SDS polyacrylamide gels (PAGE). The intensity of the Coomassie-blue stained bands was quantified by densitometry and calculated in relation to band3 [51,53].

### 4.7. NGS

Genomic DNA was isolated from peripheral EDTA blood by the QIAamp DNA Mini Kit (Qiagen GmbH, Hilden, Germany). NGS library was prepared after tagmentation with KAPA HyperPlus Kit (Roche, Mannheim, Germany). Targeted gene capture was performed using SeqCap EZ MedExome probes with subsequent target enrichment and clean-up (Roche). Pooled samples were paired-end sequenced (2 × 150 cycles) on a NextSeq500 instrument (Illumina, San Diego, CA, USA).

Sequence data were aligned to the human reference genome (NCBI genome assembly GRCh37.p13) by the NextGene software v. 2.4.2.3 (SoftGenetics, State College, PA, USA). Called variants were filtered in a first step in a subset of five spherocytosis-related genes (*ANK1* (NM_000037.4), *EPB42* (NM_000119.3), *SLC4A1* (NM_000342.3), *SPTA1* (NM_003126.2), *SPTB* (NM_001024858.2)), and in a second step followed by a subset of 117 genes using an in-house bioinformatic pipeline. The gene list consists of 117 genes that are either associated with anaemia or encode gene products that influence erythrocyte and heme metabolism. The selection process based on recommendations of other authors (e.g., [49]) and databases (e.g., OMIM, a database linking genes to human disorders; https://omim.org, accessed on 31 May 2019), but also adapted to the diagnostic requirements of the medical centre. The gene list is presented in the supplemental material, referring to NCBI gene accession numbers and OMIM entries (Table S5). The dignity of the variants was assessed according to the ACMG/AMP guidelines [23]. This scoring system is used to assign sequence variants for Mendelian disorders to five classes ranging from benign (class 1) over uncertain significance (class 3) to pathogenic (class 5). Criteria for the classification include the relevance of the gene for the underlying disease (Have variants in these genes already been described to cause the disease?), the consequence of the variant (e.g., a premature stop), the frequency of the variant in the healthy population (Pathogenic variants are rare in healthy individuals.) and the segregation of the variant with the disease within a family. The variant assessment by in silico prediction tools (NNSplice, HSF3.0, SIFT, PolyPhen2 and MutationTaster2021) [54,55,56,57,58] is also part of the evaluation process.

All pathogenic variants were confirmed by bidirectional Sanger sequencing (Sciex, Framingham, MA, USA). The detected variants were also confirmed/excluded in the patients’ relatives by Sanger sequencing. Primer sequences for PCR and cycle sequencing are available upon request.

### 4.8. Prevalence Estimation Using GnomAD

Using GnomAD (https://gnomad.broadinstitute.org/, accessed on 19 October 2023; version 3.1.2 (V3 (non-v2)) and 2.1.1 (V2)) and the Hardy-Weinberg equilibrium [59,60], we estimated the prevalence of monogenic HS in the European (non-Finnish) population. For this purpose, variants of the five genes mainly responsible for HS (*ANK1* (ENST00000289734), *EPB42* (ENST00000648595.1 (V3), ENST00000300215.3 (V2)), *SLC4A1* (ENST00000262418), *SPTA1* (ENST00000643759.2 (V3), ENST00000368147.4 (V2), *SPTB* (ENST00000389722)) were included. Due to the variable dignity of missense variants, only truncating variants (nonsense, frameshift, canonical splice site) were considered for calculation. These were supplemented in *SLC4A1* by the Bicêtre I (rs1398477044) and in *SPTA1* by αLEPRA (rs200830867). A dominant inheritance pattern was assumed for *ANK1*, *SLC4A1* and *SPTB*, and a recessive one for *SPTA1* and *EPB42*.

## 5. Conclusions

Genetic diagnosis of RBC membrane disorders is relevant for therapy and counselling of patients. When patients with haemolytic anaemia are evaluated with stringent but simple standard tests that can be offered by most laboratories, the read-out of a small, five-gene NGS panel is sufficient to confirm the diagnosis of HS. Complex tests, which are only provided by a few laboratories, can be reduced and might only be considered in rare cases.

## Figures and Tables

**Table 1:** Summary of the results of genetic and functional testing of all families. The genotype and the derived inheritance pattern are listed for all affected individuals. Spherocytes were seen in peripheral blood smear and/or deduced from a lowered mean corpuscular volume (MCV) and increased mean corpuscular haemoglobin concentration (MCHC). Reticulocytosis, lowered haptoglobin, elevated lactate dehydrogenase (LDH), and (unconjugated) bilirubin were assessed as signs of haemolysis. The constellation of laboratory results (including genotype) leads to the listed phenotype. All laboratory results are listed in detail in Supplemental Table S3. The identifiers of all index patients are listed in bold, identifiers of all affected individuals are coloured. AD = autosomal dominant, AR = autosomal recessive, d = day, F = female, M = male, m = month, wt = wildtype, y = year.

Family ID		Genotype	Inheritance	Spherocytes	Haemolysis	Phenotype/MIM Number
1	**1-1 Index (4 m, F)**	*ANK1*: c.[1405-9G>A];[=]	AD; de novo	yes	yes	Spherocytosis, type 1/# 182900
	1-2 Index case’s father (34 y, M)	wt		no		not affected
	1-3 Index case’s mother (33 y, F)	wt		no		not affected
2	**2-1 Index (20 m, F)**	*SLC4A1*: c.[2368G>C];[=], p.[(Gly790Arg)];[=]	AD	yes	yes	Spherocytosis, type 4/# 612653
	2-2 Index case’s father (39 y, M)	wt		no		not affected
	2-3 Index case’s mother (37 y, F)	*SLC4A1*: c.[2368G>C];[=], p.[(Gly790Arg)];[=]	AD	sporadic		Spherocytosis, type 4/# 612653
3	**3-1 Index (17 y, M)**	*SPTA1*: c.[7082T>C];[6531-12C>T; 5572C>G], p.[(Phe2361Ser)];[αLELY]	AR	sporadic	yes	Pyropoikilocytosis/# 266140
	3-2 Index case’s brother (20 y, M)	*SPTA1*: c.[7082T>C];[=], p.[(Phe2361Ser)];[=]		no	no	Elliptocytosis, type 2/# 130600 (carrier)
4	**4-1 Index (1 d, M)**	*SLC4A1*: c.[1468C>T];[=], p.[Arg490Cys];[=] (Bicêtre I)	AD	yes	yes	Spherocytosis, type 4/# 612653
	4-2 Index case’s father (32 y, M)	*SLC4A1*: c.[1468C>T];[=], p.[Arg490Cys];[=] (Bicêtre I)	AD			Spherocytosis, type 4/# 612653
	4-3 Index case’s mother (26 y, F)	wt		no		not affected
	4-4 Index case’s sister (7 m, F)	*SLC4A1*: c.[1468C>T];[=], p.[Arg490Cys];[=] (Bicêtre I)	AD	yes	yes	Spherocytosis, type 4/# 612653
5	**5-1 Index (3 m, F)**	*SPTB*: c.[3895delG];[=], p.[(Asp1299Metfs)];[=]	AD	yes	yes	Spherocytosis, type 2/# 616649
	5-2 Index case’s father (28 y, M)	*SPTB*: c.[3895delG];[=], p.[(Asp1299Metfs)];[=]	AD	yes	yes	Spherocytosis, type 2/# 616649
	5-3 Index case’s mother (30 y, F)	wt		no		not affected
	5-4 Index case’s brother (1 d, M)	*SPTB*: c.[3895delG];[=], p.[(Asp1299Metfs)];[=]	AD	yes	yes	Spherocytosis, type 2/# 616649
6	**6-1 Index (2 m, M)**	*SPTA1*: c.[4490G>A; 6531-12C>T];[=], p.[?];[=], *PIEZO1*: c.[6328C>T];[=], p.[(Arg2110Trp)];[=]	digenic?	yes	yes	Spherocytosis phenotype
	6-2 Index case’s father (37 y, M)	*SPTA1*: c.[4490G>A; 6531-12C>T];[6531-12C>T], p.[?];[αLELY]		yes	yes	Spherocytosis, type 3/#270970
	6-3 Index case’s mother (31 y, F)	*PIEZO1*: c.[6328C>T];[=], p.[(Arg2110Trp)];[=]		no		Stomatocytosis/#194380
7	**7-1 Index (14 m, M)**	*ANK1*: c.[2638-1G>A];[=]	AD	yes	yes	Spherocytosis, type 1/# 182900
	7-2 Index case’s mother (46 y, F)	wt		no		not affected
8	**8-1 Index (18 y, F)**	*SLC4A1*: c.[1468C>T];[=], p.[Arg490Cys];[=] (Bicêtre I)	AD	yes	yes	Spherocytosis, type 4/# 612653
9	**9-1 Index (8 y, F)**	*ANK1*: c.[5383C>T];[=], p.[(Gln1795Ter)];[=]	AD	yes	yes	Spherocytosis, type 1/# 182900
10	**10-1 Index (6 y, M)**	SPTB: c.[4266+5G>C];[=]	AD	yes	yes	Spherocytosis, type 2/# 616649
11	**11-1 Index (11 y, F)**	*ANK1*: c.[4306C>T];[=], p.[(Arg1436Ter)];[=]	AD; de novo	yes	yes	Spherocytosis, type 1/# 182900
	11-2 Index case’s father (45 y, M)	wt		no	no	not affected
	11-3 Index case’s mother (48 y, F)	wt		no	no	not affected
12	**12-1 Index (9 y, F)**	*SPTB*: c.[4759C>T];[=], p.[(Gln1587Ter)];[=]	AD; de novo	yes	yes	Spherocytosis, type 2/# 616649
	12-2 Index case’s father (50 y, M)	wt		no		not affected
	12-3 Index case’s mother (48 y, F)	wt		no		not affected
13	**13-1 Index (8 y, F)**	*SPTB*: c.[c.4873C>T];[=], p.[(Arg1625Ter)];[=]	AD	yes	yes	Spherocytosis, type 2/# 616649
14	**14-1 Index (10 y, M)**	*ANK1*: c.[457C>T];[=], p.[(Gln153Ter)];[=]	AD	yes	yes	Spherocytosis, type 1/# 182900
	14-2 Index case´s sister (3 d, F)	*ANK1*: c.[457C>T];[=], p.[(Gln153Ter)];[=]	AD	yes	yes	Spherocytosis, type 1/# 182900
	14-3 Index case’s mother (37 y, F)	wt		no		not affected
15	**15-1 Index (6 y, M)**	*SPTA1*: c.[1677+1G>A;6531-12C>T; 5572C>G](;)[4339-99C>T], p.[(?)](;)[αLEPRA]	AR	yes	yes	Spherocytosis, type 3/# 270970
16	16-1 Index (6 y, F)	*SPTA1*: c.[5386C>T];[6531-12C>T], p.[(Gln1796Ter)];[αLELY], *EPB42*: c.[827G>A];[=], p.[(Arg276Gln)];[=]	digenic?	sporadic	yes	Spherocytosis phenotype
	16-2 Index case’s father (38 y, M)	*SPTA1*: c.[5386C>T];[6531-12C>T], p.[(Gln1796Ter)];[αLELY]		no	yes	Spherocytosis, type 3?/# 270970
17	**17-1 Index (3 y, F)**	*ANK1*: c.[1282dup];[=], p.[(Ala428fs)];[=]	AD; de novo?	yes	yes	Spherocytosis, type 1/# 182900
18	**18-1 Index (17 y, F)**	wt		inconclusive	yes	not affected

**Table 2:** Classification of the causative variants. We detected 20 pathogenic or likely pathogenic variants in our patients. Eight of these variants (including the low expression alleles αLELY and αLEPRA) have already been described, while 12 of 20 variants are reported here for the first time. All variants were analysed using the in silico tools NNSplice and HSF 3.0 or PolyPhen2, SIFT and MutationTaster 2021. New variants were classified according to ACMG/AMP criteria. MAF = minor allele frequency, NMD = nonsense-mediated decay, PTC = premature termination codon, x = no data available/not applicable.

**New Variants**													
***Gene***	**RefSeq Number**	**Variant**	**MAF (dbSNP)**	**rsID (dbSNP)**		**Bioinformatic Analyses**						**Predicted** **Consequences** **on Protein Level**	**Classification** **(ACMG/AMP)**
						**NNSplice/HSF 3.0**			**Poly Phen2**	**SIFT**	**Mutation Taster**		
*ANK1*	NM_000037.4	c.457C>T, p.(Gln153Ter)	x	x		x			x	x	deleterious	PTC, NMD assumed	pathogenic
*ANK1*	NM_000037.4	c.1282dup, p.(Ala428fs)	0.00005	rs769735016		x			x	x	deleterious	PTC, NMD assumed	pathogenic
*ANK1*	NM_000037.4	c.2638-1G>A	x	x		broken acceptor site			x	x	deleterious	Splice defect	pathogenic
*ANK1*	NM_000037.4	c.5383C>T, p.(Gln1795Ter)	x	x		x			x	x	deleterious	PTC, NMD assumed	pathogenic
*SLC4A1*	NM_000342.3	c.2368G>C, p.(Gly790Arg)	x	x		x			probably damaging, score 1	deleterious, score 0	deleterious	Amino acid substitution	likely pathogenic
*SPTA1*	NM_003126.2	c.1677+1G>A	0.000007	rs1653872984		broken donor site			x	x	deleterious	PTC, NMD assumed	pathogenic
*SPTA1*	NM_003126.2	c.7082T>C, p.(Phe2361Ser)	x	x		x			probably damaging, score 0.937	deleterious, score 0	deleterious	Amino acid substitution	likely pathogenic
*SPTB*	NM_001024858.2	c.3895delG, p.(Asp1299Metfs)	x	x		x			x	x	deleterious	PTC, NMD assumed	pathogenic
*SPTB*	NM_001024858.2	c.4266+5G>C	as G>A (0.000007)	rs1208350542		broken donor site			x	x	benign	Splice defect	likely pathogenic
*SPTB*	NM_001024858.2	c.4759C>T, p.(Gln1587Ter)	as C>G (0.00000)	rs2082338281		x			x	x	deleterious	PTC, NMD assumed	pathogenic
*EPB42*	NM_000119.3	c.827G>A, p.(Arg276Gln)	0.00003	rs927148032		x			probably damaging, score 0.933	tolerated, score 0.36	deleterious	Amino acid substitution	likely pathogenic
*PIEZO1*	NM_001142864.4	c.6328C>T, p.(Arg2110Trp)	0.000006	rs776531529		x			probably damaging, score 0.994	deleterious, score 0	deleterious	Amino acid substitution	likely pathogenic
**Known Variants**													
***Gene***	**RefSeq Number**	**Variant**	**ClinVar** **Allele ID**	**MAF (dbSNP)**	**rsID (dbSNP)**		**Bioinformatic Analyses**					**Predicted** **Consequences** **on Protein Level**	**Reference**
							**NNSplice/** **HSF 3.0**	**Poly Phen2**		**SIFT**	**Mutation Taster**		
*ANK1*	NM_000037.4	c.1405-9G>A	x	x	x		broken acceptor site	x		x	benign	Splice defect	[27,28]
*ANK1*	NM_000037.4	c.4306C>T, p.(Arg1436Ter)	799540 (pathogenic)	x	rs1586072383		x	x		x	deleterious	PTC, NMD assumed	[29]
*SLC4A1*	NM_000342.3	Bicêtre I: c.1468C>T, p.(Arg490Cys)	x	0.00007	rs1398477044		x	probably damaging, score 1		damaging, score 0	deleterious	Amino acid substitution	[30]
*SPTA1*	NM_003126.2	αLEPRA: c.4339-99C>T	973143 (pathogenic)	0.004	rs200830867		no effect	x		x	deleterious	Splice defect	[31,32]
*SPTA1*	NM_003126.2	c.4490G>A, p.(Gly1497Glu)	249441 (conflicting)	0.017	rs41273523		x	tolerated		benign	benign	Amino acid substitution	not listed in cis with αLELY or described in the literature
*SPTA1*	NM_003126.2	c.5386C>T, p.(Gln1796Ter)	981247 (pathogenic)	0.000008	rs763899069		x	x		x	deleterious	PTC, NMD assumed	not described in the literature
*SPTA1*	NM_003126.2	αLELY: c.(5572C>G; 6531-12C>T)	249434/ 249428 (conflicting)	0.28263/0.21386	rs3737515/ rs28525570		no effect	x		x	benign	Splice defect	[33]
*SPTB*	NM_001024858.2	c.4873C>T, p.(Arg1625Ter)	1451194 (pathogenic)	x	x		x	x		x	deleterious	PTC, NMD assumed	[3]

## Data Availability

All relevant data generated or analysed during this study are included in this published article or its Supplementary Materials. Restrictions apply to the availability of the complete next-generation sequencing data of the patients to preserve patient confidentiality. The corresponding author will on request detail the restrictions and any conditions under which access to some data may be provided.

## Supplementary Materials

The supporting information can be downloaded at: https://www.mdpi.com/article/10.3390/ijms242317021/s1. References [8,35,60,61,62,63,64,65,66,67,68] are cited in the supplementary materials.
