# Supplementary material for: Hereditary Spherocytosis: Can Next-Generation Sequencing of the Five Most Frequently Affected Genes Replace Time-Consuming Functional Investigations?
Source: Int J Mol Sci. 2023 Nov 30;24(23):17021. doi: 10.3390/ijms242317021 (PMC10707146; doi:10.3390/ijms242317021)
Supplement: Supplementary file 1 [file ijms-24-17021-s001.zip › Suppl_Figures.pdf]

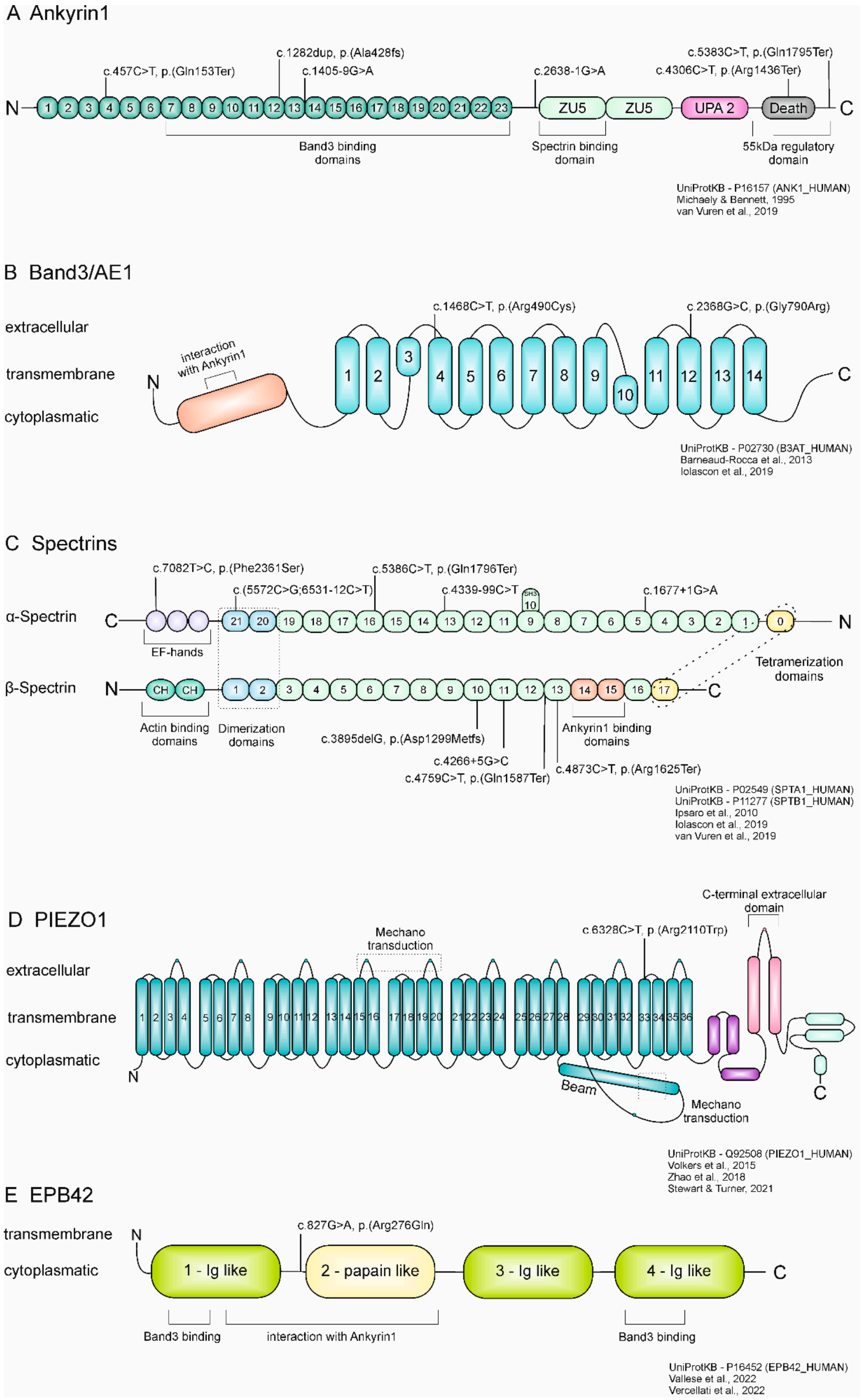

Figure S1. Schematic representation of the affected gene products in our collective. The detected pathogenic or likely pathogenic variants are assigned in a simplified scheme of the proteins. Splice variants are indicated next to the closest amino acid. The effect of the splice defect is not illustrated. The structure of the proteins is based on Uniprot entries

(https://www.uniprot.org/, accessed on 25 May 2023) and data from literature. (**A**) Ankyrin (*Ank1*) [*1, 2*], (**B**) Band3 (*SLC4A1*) [*3, 4*], (**C**) α- and β-spectrin (*SPTA1, SPTB*) [*2, 4, 5*], (**D**) PIEZO 1 (*PIEZO1*) [*6–8*], (**E**) Band4.2 (*EPB42*) [*9, 10*]

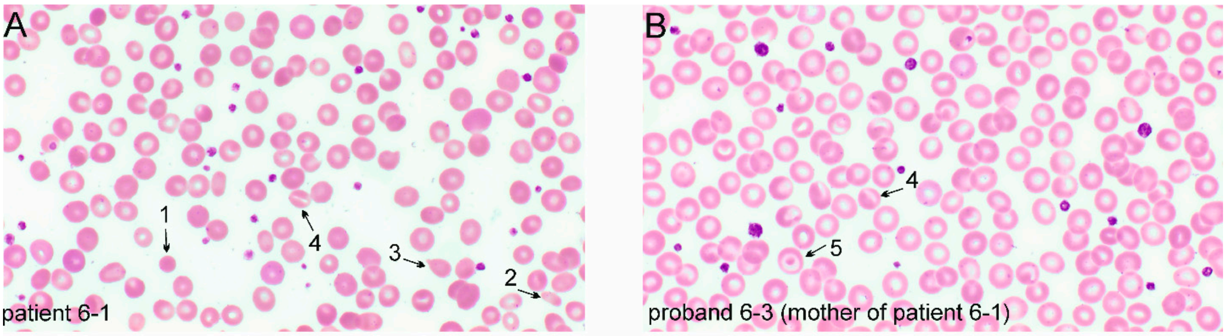

Figure S2. RBC morphology (May-Grunwald-Giemsa stain of peripheral blood smears) of two genetically tested individuals. The erythrocyte morphology is in accordance with the phenotype expected from the gene defect. (**A**) In patient 6-1, stomatocytes as well as spherocytes were present. Besides the *SPTA1* variant of the father, the mother's *PIEZO1* variant probably influences the haemolytic phenotype of the patient (digenic inheritance). (**B**) In his mother, proband 6-3, who has no clinical symptoms of haemolysis, stomatocytes and target cells were detected. The occurrence of stomatocytes supports the classification of the proband's *PIEZO1* variant as relevant (gain of function variant in dehydrated stomatocytosis). Cell types, as indicated by arrow: 1: microspherocyte, 2: elliptocyte, 3: dacrocyte (tear drop cell), 4: stomatocyte, 5: codocyte (target cell)

References

1. Michaely, P.; Bennett, V. The ANK repeats of erythrocyte ankyrin form two distinct but cooperative binding sites for the erythrocyte anion exchanger. *The Journal of biological chemistry* **1995,** *270* (37), 22050–22057. DOI: 10.1074/jbc.270.37.22050.
2. van Vuren, A.; van der Zwaag, B.; Huisjes, R.; Lak, N.; Bierings, M.; Gerritsen, E.; van Beers, E.; Bartels, M.; van Wijk, R. The Complexity of Genotype-Phenotype Correlations in Hereditary Spherocytosis: A Cohort of 95 Patients: Genotype-Phenotype Correlation in Hereditary Spherocytosis. *HemaSphere* **2019,** *3* (4), e276. DOI: 10.1097/HS9.0000000000000276.
3. Barneaud-Rocca, D.; Etchebest, C.; Guizouarn, H. Structural model of the anion exchanger 1 (SLC4A1) and identification of transmembrane segments forming the transport site. *The Journal of biological chemistry* **2013,** *288* (37), 26372–26384. DOI: 10.1074/jbc.M113.465989.
4. Iolascon, A.; Andolfo, I.; Russo, R. Advances in understanding the pathogenesis of red cell membrane disorders. *British journal of haematology* **2019,** *187* (1), 13–24. DOI: 10.1111/bjh.16126.
5. Ipsaro, J. J.; Harper, S. L.; Messick, T. E.; Marmorstein, R.; Mondragón, A.; Speicher, D. W. Crystal structure and functional interpretation of the erythrocyte spectrin tetramerization domain complex. *Blood* **2010,** *115* (23), 4843–4852. DOI: 10.1182/blood-2010-01-261396.
6. Stewart, L.; Turner, N. A. Channelling the Force to Reprogram the Matrix: Mechanosensitive Ion Channels in Cardiac Fibroblasts. *Cells* **2021,** *10* (5). DOI: 10.3390/cells10050990.
7. Volkers, L.; Mechioukhi, Y.; Coste, B. Piezo channels: from structure to function. *Pflugers Archiv : European journal of physiology* **2015,** *467* (1), 95–99. DOI: 10.1007/s00424-014-1578-z.
8. Zhao, Q.; Zhou, H.; Chi, S.; Wang, Y.; Wang, J.; Geng, J.; Wu, K.; Liu, W.; Zhang, T.; Dong, M.-Q.; Wang, J.; Li, X.; Xiao, B. Structure and mechanogating mechanism of the Piezo1 channel. *Nature* **2018,** *554* (7693), 487–492. DOI: 10.1038/nature25743.

9. Vallese, F.; Kim, K.; Yen, L. Y.; Johnston, J. D.; Noble, A. J.; Calì, T.; Clarke, O. B. Architecture of the human erythrocyte ankyrin-1 complex. *Nature structural & molecular biology* **2022,** *29* (7), 706–718. DOI: 10.1038/s41594-022-00792-w.
10. Vercellati, C.; Marcello, A. P.; Fattizzo, B.; Zaninoni, A.; Seresini, A.; Barcellini, W.; Bianchi, P.; Fermo, E. Effect of primary lesions in cytoskeleton proteins on red cell membrane stability in patients with hereditary spherocytosis. *Frontiers in physiology* **2022,** *13*, 949044. DOI: 10.3389/fphys.2022.949044.
